# WAY-100635 Alleviates Corneal Lesions Through 5-HT_1A_ Receptor-ROS-Autophagy Axis in Dry Eye

**DOI:** 10.3389/fmed.2021.799949

**Published:** 2021-12-14

**Authors:** Xujiao Zhou, Yiqin Dai, Zimeng Zhai, Jiaxu Hong

**Affiliations:** ^1^Department of Ophthalmology, Eye and Ear, Nose and Throat (ENT) Hospital, Fudan University, Shanghai, China; ^2^Department of Ophthalmology, The Affiliated Hospital of Guizhou Medical University, Guiyang, China

**Keywords:** 5-HT_1A_ receptors, reactive oxygen species, autophagy, dry eye, WAY-100635

## Abstract

**Purpose:** To explore whether 5-HT_1A_ receptors are involved in the dry eye disease (DED) mouse model and reveal its underlying mechanism.

**Methods:** A C57BL/6J mouse DED model was established via the administration of 0.2% benzalkonium chloride twice a day for 14 days. Corneal fluorescein sodium staining score and Schirmer I test were checked before, and on days 7, 14, and 21 after treatment. The experiment was randomly divided into control, DED, 5-HT_1A_ receptor agonist with or without N-acetylcysteine (NAC) and 5-HT_1A_ receptor antagonist with or without NAC groups. The mRNA expression of inflammatory cytokines was measured by reverse transcription-quantitative polymerase chain reaction. Cellular reactive oxygen species (ROS) were detected by 2', 7'-dichlorodihydrofluorescein diacetate assays. Western blot analysis was used to measure the expression levels of autophagic proteins microtubule-associated protein 1 light chain 3 (LC3B-I/II) and autophagy-related gene 5 (ATG5).

**Results:** 5-HT_1A_ receptor agonist (8-OH-DPAT) increased corneal fluorescein sodium staining spots and 5-HT_1A_ receptor antagonist (WAY-100635) decreased them. Treatment with 8-OH-DPAT was associated with the gene expression of more inflammatory cytokines, such as interleukin-6 (IL-6), tumor necrosis factor-α (TNF-α), C-C motif chemokine ligand 2 (CCL2) and C-X-C motif chemokine ligand 10 (CXCL10) compared with treatment with WAY-100635. An increased expression of LC3B-I/II and ATG5 was observed in corneal epithelial cells in the mouse model of DED. 8-OH-DPAT significantly enhanced the expression of LC3B-I/II and ATG5 by disrupting ROS levels. WAY-100635 alleviates autophagy by inhibiting ROS production.

**Conclusion:** Excessive ROS release through 8-OH-DPAT induction can lead to impaired autophagy and increased inflammatory response in DED. WAY-100635 reduces corneal epithelial defects and inflammation in DED, as well as alleviates autophagy by inhibiting ROS production. The activation of the 5-HT_1A_ receptor-ROS-autophagy axis is critically involved in DED development.

## Introduction

Dry eye disease (DED) is a chronic and multifactorial ocular surface disease that affects 5–35% of the worldwide population, with its prevalence increasing with age (1). Patients with DED suffer from ocular discomfort, which affects their vision-related quality of life (2). Increased tear film hyperosmolarity and instability lead to the damage of the corneal epithelium through a cascade of inflammatory events, inducing neurosensory abnormalities (2, 3).

Patients with DED exhibit a tendency toward a depressive mood. However, the treatment of depression with selective serotonin (5-hydroxytryptamine; 5-HT) reuptake inhibitors induces the secretion of proinflammatory cytokines, and cell apoptosis on the ocular surface aggravates depression-associated DED by activating the nuclear factor-κB (NF-κB) pathway (4). 5-HT is an important endogenous monoamine neurotransmitter and neuromodulator (5). The physiological and pharmacological mechanisms of 5-HT function mainly through seven receptor families, 5HT_1_-5HT_7_ (6). Therefore, the 5-HT_1A_ receptor gene is a strong candidate for the treatment of depression, as it has been shown to inhibit depression symptoms in 5-HT_1A_ receptor knockout mouse models (7–9).

As part of the outermost layer of the eye, corneal epithelial cells are susceptible to a variety of environmental stimuli. Excessive reactive oxygen species (ROS) can cause cellular imbalance in oxidative stress and induce human corneal epithelial cell (HCECs) injury by targeting DNA, proteins and intracellular processes (10). ROS may function as indispensable signaling molecules in the activation of autophagy (11). ROS can lead to autophagosome accumulation, ultimately mediating non-apoptotic cell death (12, 13). In a previous study, autophagy occurred in the lacrimal glands of the mouse model of DED; a significant colocalization of LC3-phosphatidylethanolamine conjugate (LC3B) and autophagy-related gene 5 (ATG5) was detected in the salivary glands of patients with primary Sjögren's syndrome (14). 5-HT_1A_ receptors have the potential to regulate oxidative stress in the retina and retinal pigment epithelium. The activation of these receptors is coupled with neuronal and non-neuronal intracellular signaling pathways (15). However, to date, the role and regulatory mechanism of 5-HT_1A_ receptors in autophagy remain undefined in DED.

The present study focused on exploring whether a 5-HT_1A_ receptor antagonist can effectively resist autophagy in the DED model. Our results revealed an increased expression of 5-HT_1A_ receptors along with an overproduction of ROS and impaired autophagy in DED. The inhibition of 5-HT_1A_ receptors reduces excessive ROS release and enhances autophagic flux, thereby inhibiting corneal epithelial cell apoptosis in DED mice. To the best of our knowledge, this is these findings are the first to show that the 5-HT_1A_ receptor-ROS-autophagy axis is critically involved in DED development.

## Materials and Methods

### DED Model and Treatment

All experimental protocols complied with the Association for Research in Vision and Ophthalmology Staement for the use of animals and were approved by the Fudan University Ethics Committee (ethical code: EENTIRB-2018-03-01). A total of 50 male C57BL/6J mice aged 6–8 weeks old were purchased from SLAC Laboratory Animal Co., Ltd. (Shanghai, China). Mice were housed in an environmentally controlled room with 60% humidity under a 12/12 h light-dark cycle. Mouse eyes were administrated with 5 μL of 0.2% benzalkonium chloride twice a day for 14 consecutive days, while pphosphate-bufferedsaline was used to treat the control group. Corneal fluorescein sodium staining and Schirmer I test scores were recorded before, and at days 7, 14, and 21 after treatment. The mice were randomly divided into experimental group 1 (receiving a subconjunctival injection of 8-OH-DPAT), experimental group 2 (receiving a subconjunctival injection of WAY-100635), experimental group 3 [receiving a subconjunctival injection of N-acetylcysteine (NAC)], experimental group 4 (receiving a subconjunctival injection of NAC + 8-OH-DPAT), experimental group 5 (receiving a subconjunctival injection of NAC + WAY-100635), experimental DED group (receiving a subconjunctival injection of phosphate-buffered saline) and a blank control group.

### Corneal Fluorescein Sodium Staining Score

A total of 1 μL of 1% sodium fluorescein was instilled into the lateral conjunctival sac to evaluate the degree of corneal epithelium defects in the mice. The corneal epithelial integrity was visualized using a cobalt blue filter under a slit lamp microscope. Each cornea was divided into 4 quadrants that were scored individually using the following four-point scale (0–4): 0 points, no staining; 1 point, <30 stained dots; 2 points, >30 non-diffuse stained dots; 3 points, severe diffuse staining but no plaque staining; 4 points, positive fluorescein plaque. The scores from each quadrant were summed to obtain the final score (0–16).

### Detection of Basal Tear Secretion (Schirmer I Test)

Phenol-Red thread tear test (Schirmer I) was used with Zone-Quick (Showa Yakuhin Kako Co., Ltd). Following general anesthesia with 1.25% avertin administered intraperitoneally, 1 mm of the folded end of the test strip was inserted into the lateral upper conjunctival fornix for 20 s. The color of phenol cotton was yellow (acidic) and changed from yellow to red when it came into contact with tears. Red dye the wetted length (mm) of the test strip, which was recorded.

### RNA Extraction and Reverse Transcription-Quantitative Polymerase Chain Reaction Analysis

According to the manufacturer's instructions, total RNA was extracted from mouse corneal tissue using TRIzol reagent (Invitrogen; Thermo Fisher Scientific, Inc., Waltham, MA, USA). The RNA sample was then reverse-transcribed into cDNA using the PrimeScript RT reagent kit (Takara Bio, Inc. Otsu, Japan). RT-qPCR analysis was performed using SYBR Premix Ex Taq (Takara Bio, Inc.) with an ABI Prism 7500 sequence detection system (Applied Biosystems; Thermo Fisher Scientific, Inc.). The relative expression of IL-1β, IL-6, CCL2 and CXCL10 was normalized to the endogenous control, GAPDH, using the 2^−ΔΔCt^ method.

### Western Blot Analysis

Mouse corneal tissues were lysed using a radioimmunoprecipitation assay solution (Beyotime Institute of Biotechnology, Shanghai, China), and the protein concentration was determined using the bicinchoninic acid assay (Beyotime Institute of Biotechnology). Proteins were electrotransferred onto 0.45 μm polyvinylidene fluoride membranes (Immobilon-P; MilliporeSigma, Burlington, MA, USA). Membranes were blocked with 5% non-fat dry milk for 2 h at room temperature and incubated with the following primary antibodies: mouse monoclonal antibodies against LC3B (dilution, 1:1000; catalog no. 83506; Cell Signaling Technology, Inc., Danvers, MA, USA) and rabbit monoclonal antibodies against ATG5 (dilution, 1:1000; catalog no. ab108327; Abcam). The membranes were incubated with peroxidase-conjugated secondary antibodies, and β-actin was used as a loading control.

### ROS Detection

The fluorescence detection kit used to detect ROS in frozen sections produces fluorescence under conditions of oxidative tissue damage through the transmembrane fluorescent dye dichloro-fluoro-acetoacetate and quantitatively detects the existence of ROS groups in frozen tissues. 2′, 7′-Dichlorofluorescein diacetate is a coloring agent whose membrane is completely free. Once it is oxidized by hydrogen peroxide, peroxide groups, peroxynitrite anions, etc., fluorescence is generated. The ROS concentration in tissue cells was measured using that method.

### Statistical Analysis

Quantitative data are expressed as the mean ± standard error of the mean. One-way analysis of variance was used for differences among multiple groups, and the Bonferroni test for differences between two groups. Student's *t*-test was used to identify differences between groups. *P* < 0.05 was considered to indicate a statistically significant difference.

## Results

### WAY-100635 Can Alleviate Corneal Epithelial Cell Injury in the DED Model

**Figure 1:**
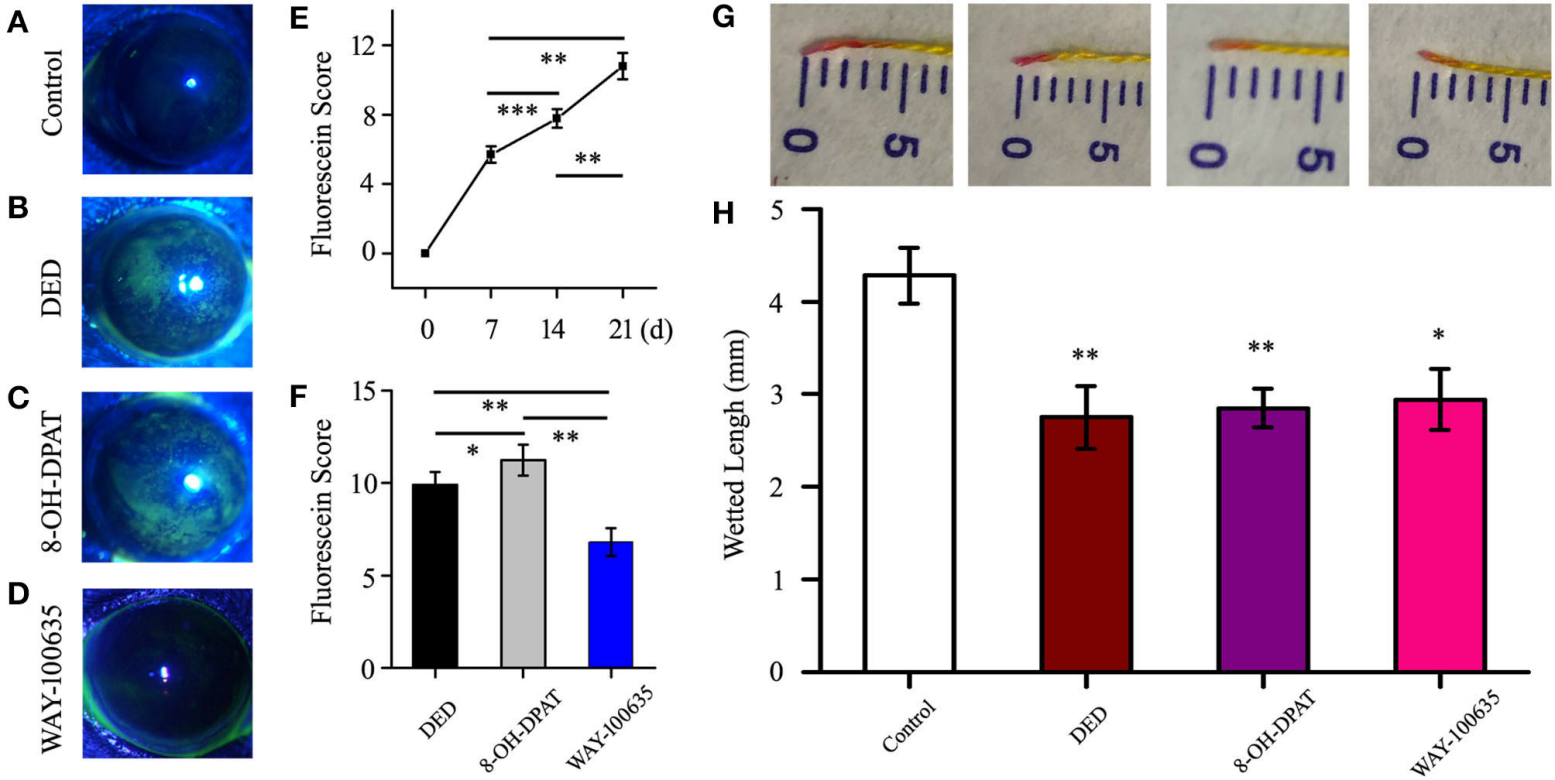
Imaging and scoring following treatment with benzalkonium chloride twice a day. **(A-D)** Representative examples for each scale of fluorescence staining. Following successful modeling, the fluorescence staining points on the corneal epithelium increased in density, and the serious sites were plaques. **(E)** Time-score curve of the fluorescein sodium scores at 7, 14 and 21 days after modeling. **(F)** Statistical chart of corneal fluorescein staining scores in the DED, 8-OH-DPAT and WAY-100635 groups. **(G)** Representative photos for each scale of phenol red thread test. **(H)** On day 21, the amount of tear secretion in the DED group was significantly reduced, but no treatment was effective. ^*^P < 0.05, ^**^P < 0.01 and ^***^P < 0.001. DED, dry eye disease.

A special 5-HT_1A_ receptor agonist (8-OH-DPAT) and antagonist (WAY-100635) was used to verify the role of 5-HT_1A_ receptors in DED mice. Staining in the DED mouse model was shown to be diffuse and progressively intense in the 8-OH-DPAT treatment groups (Figures 1A–C), and a general trend of increased corneal fluorescein staining scores was observed, as compared with those of the controls (Figure 1D). The staining scores of sodium fluorescein on days 7, 14 and 21 were 5.7 ± 0.48, 7.78 ± 0.53 and 10.8 ± 0.76, respectively. The staining scores of corneal fluorescein were higher in 8-OH-DPAT-treated mice (11.23 ± 0.84) than in WAY-100635-treated mice (6.8 ± 0.75, *P* < 0.01) and mice in the DED groups (9.89 ± 0.71, *P* < 0.05) (Figure 1E). Tear secretion was significantly reduced in DED mice (2.75 ± 0.34 vs. 4.28 ± 0.3 mm in the control group). However, no differences in tear secretion were observed among the DED, 8-OH-DPAT and WAY-100635 groups (Figures 1F,G). 8-OH-DPAT treatment induced a typical corneal epithelial defect and WAY-100635 reduced corneal epithelial injury.

### WAY-100635 Reduces Inflammatory Response

**Figure 2:**
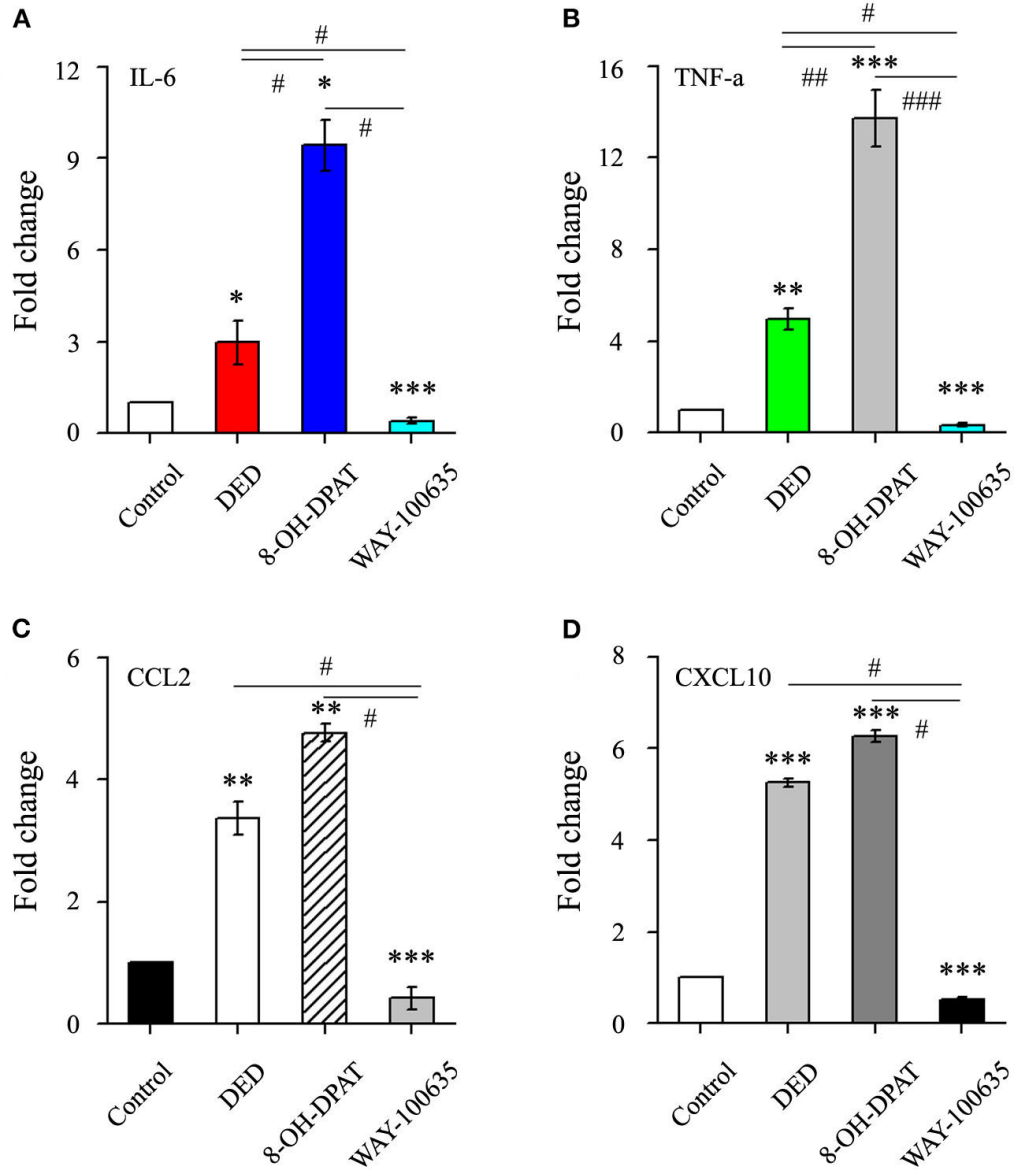
The effects of 8-OH-DPAT on the inflammatory response were blocked by the administration of WAY-100635. **(A)** Quantification of IL-6 in control and DED corneas following treatment with 8-OH-DPAT and WAY-100635. **(B)** Quantification of TNF-α in control and DED corneas following treatment with 8-OH-DPAT and WAY-100635. **(C)** Quantification of CCL2 in DED corneas of eyes treated with 8-OH-DPAT and WAY-100635, compared with the controls. **(D)** Quantification of CXCL10 in DED corneas of eyes treated with 8-OH-DPAT and WAY-100635, compared with the controls. ^*^P < 0.05, ^**^P < 0.01, and ^***^P < 0.001 vs. control; ^#^*P* < 0.05, ^##^*P* < 0.01, and ^###^*P* < 0.001 among DED, 8-OH-DPAT and WAY-100635 groups (one-way analysis of variance). DED, dry eye disease; TNF-α, tumor necrosis factor-α; CCL2, C-C motif chemokine ligand 2; CXCL10, C-X-C motif chemokine ligand 10.

The inflammatory cytokine levels in corneal tissues were further analyzed. The RT-qPCR assay results demonstrated that the gene expression of inflammatory cytokines IL-6, TNF-α, CCL2 and CXCL10 were increased in the DED group compared to those of the control group. Treatment with 8-OH-DPAT was associated with more inflammatory cytokines and chemokines than treatment with WAY-100635 (9.43 ± 0.84 vs. 0.4 ± 0.09 in IL-6, *n* = 6, *P* < 0.05; 13.74 ± 1.25 vs. 0.33 ± 0.09 in TNF-α, *n* = 5, *P* < 0.001; 4.77 ± 0.14 vs. 0.42 ± 0.19 in CCL2, *n* = 7, *P* < 0.05; 6.27 ± 0.12 vs. 0.52 ± 0.05 in CXCL10, *n* = 13, *P* < 0.05; Figure 2). The results suggested that 8-OH-DPAT aggravates inflammatory damage in DED corneal epithelium and WAY-100635 alleviates it.

### WAY-100635 Decreases ROS Production

**Figure 3:**
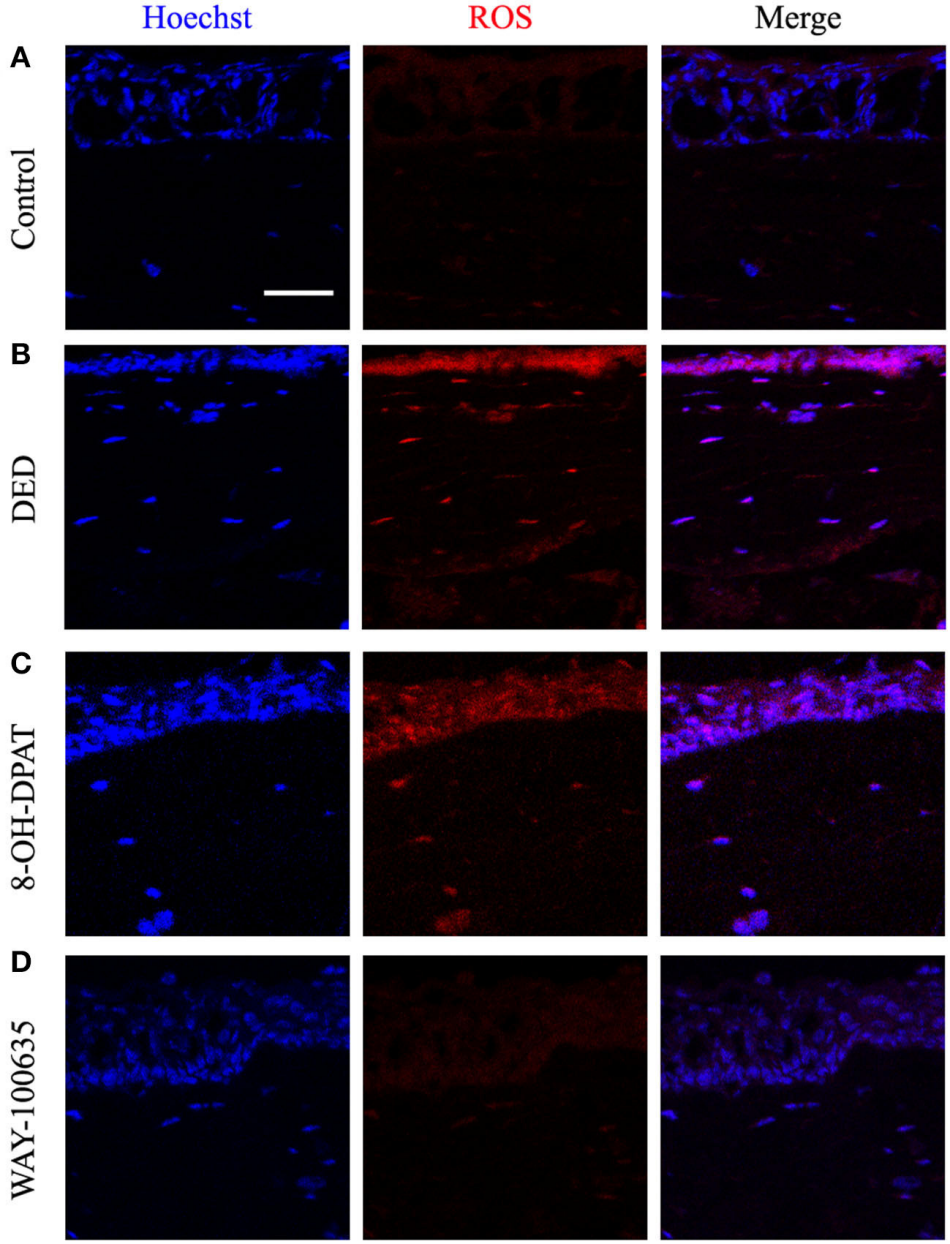
Effects of 8-OH-DPAT and WAY-100635 on ROS levels. **(A)** Micrographs of 10-μm thick transverse sections of control corneas. **(B)** Representative confocal microscope image from the DED groups. **(C,D)** Confocal micrographs of 10-μm thick transverse sections of control corneas between the 8-OH-DPAT and WAY-100635 groups. Bar, 50 μm. DED, dry eye disease; ROS, reactive oxygen species.

Previous studies have indicated that ROS production is accompanied by inflammatory activation (16, 17). Therefore, in order to verify the role of ROS in DED with or without drug treatment, changes in ROS levels were measured in frozen sections of corneal tissue. Representative confocal micrographs showed that 8-OH-DPAT increased ROS production, while pretreatment with WAY-100635 markedly decreased DED-induced ROS (Figure 3).

### 5-HT_1A_ Receptors Regulate Autophagy Through the ROS Pathway

**Figure 4:**
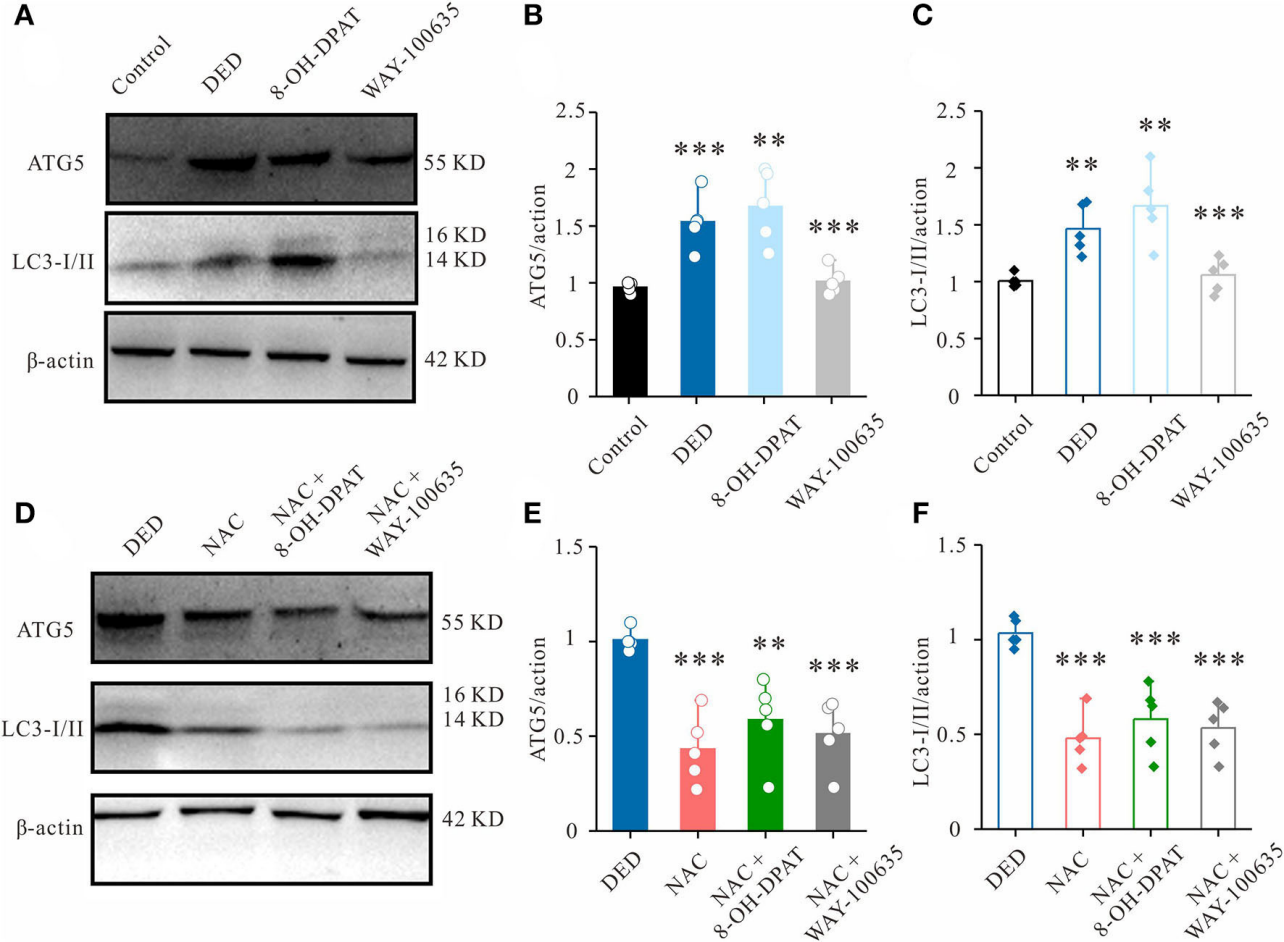
Effects of 8-OH-DPAT and WA-100635 on ROS levels. **(A)** The expression of autophagy markers ATG5 and LC3-I/II in the cornea was measured by western blot analysis. **(B,C)** Relative densitometry quantification of **(B)** ATG5 and (**C**) LC3-I/II. **(D)** Western blot analysis results showing the changes in ATG5 and LC3-I/II expression in the corneal epithelium of DED mice from the NAC-, NAC + 8-OH-DPAT- and NAC + WAY-100635-treated groups. **(E,F)** Relative densitometry quantification of **(E)** ATG5 and **(F)** LC3-I/II among groups. ^**^P < 0.01 and ^***^P < 0.001. ROS, reactive oxygen species; ATG5, autophagy-related gene 5; LC3-I/II, microtubule-associated protein 1 light chain 3; DED, dry eye disease; NAC, N-acetylcysteine.

Western blot analysis showed that the expression of the autophagic marker LC3B-I/II was significantly increased in DED mice. To further explore the role of 5-HT_1A_ receptors in DED autophagy, changes in autophagic protein expression following the activation of 5-HT_1A_ receptor and application of the inhibitor in corneal tissues were investigated. It was found that 8-OH-DPAT increased the protein expression levels of LC3B-I/II and ATG5, while WAY-100635 decreased it (Figures 4A–C).

Previous studies have revealed that oxidative stress and autophagy maintain a cross-talk regulation (18, 19). Therefore, to verify the important role of the 5-HT_1A_ receptor-ROS axis in DED autophagy, a ROS scavenger, NAC, was used. In the DED model, it was found that a subconjunctival injection of NAC could also decrease LC3B-I/II and ATG5 expression (Figures 4D–F). In the presence of NAC, the additional administration of 8-OH-DPAT did not induce LC3B or ATG5 upregulation (Figures 4D–F). In combination, these results suggested that 5-HT_1A_ receptors are crucial endogenous regulators of autophagy that exert their effects by regulating ROS in DED.

## Discussion

In the present study, 8-OH-DPAT was found to cause corneal epithelial injury in an animal model of DED. First, the corneal fluorescein sodium test results showed that 8-OH-DPAT increased the staining spots but WAY-100635 decreased them. Second, 8-OH-DPAT and WAY-100635 did not exacerbate the effects of tear secretion. Third, treatment with 8-OH-DPAT was associated with more inflammatory cytokines and chemokines than treatment with WAY-100635. Finally, WAY-100635 alleviated autophagy by inhibiting ROS production. In combination, the results of the present study revealed a novel mechanism through which 5-HT_1A_ receptor inhibition modulated the ROS-autophagy axis and thereby helped protect DED.

A cross-sectional study found a positive relationship between DED subjective symptoms and depression/anxiety scores (20). Mrugacz et al. reported that tear levels of IL-6, IL-17, and TNF-α were significantly correlated to the DED severity, which were higher in depressed patients than in the normal group (21). The reasons that DED is often found in depressed/anxious populations remain unclear. Several studies indicated a potential connection between the serotonin function and the tear secretion in the lacrimal gland (LG). In addition, Chhadva et al. reported that tear serotonin concentration positively correlated with symptoms of DED (22). Our findings were in agreement with their results.

Previous studies have found that 8-OH-DPAT attenuated morphine-induced apoptosis in the dorsal raphe nucleus of rat (23). In primary cultures of neurons from chick embryo telencephalons, 8-OH-DPAT can reduced the number of apoptotic cells in a concentration dependent manner (24). However, in our present study, the effect of 8-OH-DPAT seemed to be opposite to that of previous studies. Using a mouse model of DED, we presented the evidence that 8-OH-DPAT aggravated the injury of corneal epithelial cells, while WAY-100635 alleviated them. The reason for this discrepancy is unclear, one possible explanation for the differences in results could be related to the design of the experimental models and the different target cells used in previous studies.

Autophagy is an evolutionarily conserved, dynamic and lysosome-mediated intracellular process that works by mediating the sequestration and delivery of cytoplasmic material into the lysosome, where it is degraded and recycled (25, 26). The dysregulation of autophagy is linked to various autoimmune and auto-inflammatory diseases. Although it remains unclear whether autophagy has a toxic or protective effect on cells, accumulating evidence suggests that autophagy markers are increased in DED (27–29). LC3 is a microtubule-associated protein that is processed by cleavage (producing LC3-I) and subsequent lipid conjugation (yielding LC3-II) for membrane targeting. LC3B induction is an established marker of autophagosomes (30). Furthermore, the gene expression of ATG5 is involved in the early stages of autophagosome formation (27). Compared to the controls, ATG5 levels in tear and conjunctival epithelial cells were upregulated in Sjögren's syndrome DED but not in non-Sjögren's syndrome DED. Although clinical trials with large sample sizes would be useful to confirm these findings, ATG5 is expected to serve as a disease-specific diagnostic marker of Sjögren's syndrome DED. In this study, increased levels of LC3B-I/II and ATG5 were observed in DED both *in vivo*. The results showed that 8-OH-DPAT-5-HT_1A_ receptor activation stimulates the expression of LC3B-I/II and ATG5. These findings suggested that 5-HT_1A_ receptors may participate in the remodeling of the corneal epithelial cell microenvironment to regulate DED.

In addition, oxidative stress can be a primary cause of autophagy impairment (18, 19). Previous studies have revealed that the oxidative stress-induced inhibition of autophagy plays a pivotal role in cadmium-mediated cytotoxicity in primary rat proximal tubular cells (31). NAC, an ROS scavenger, has been investigated with respect to DED treatment (32–34). Previous studies have shown that, in HCECs subjected to hyperosmolar conditions, ROS are overproduced and mitochondrial function is impaired (10). In the present DED mouse model, it was found that NAC could reduce the expression of LC3B-I/II and ATG5 in corneal epithelium cells. To the best of our knowledge, this study was the first to suggest that NAC can protect the corneal epithelium by normalizing impaired autophagic degradation in DED.

Reports have suggested that serotonin reuptake inhibitors may aggravate DED by inducing severe inflammation and HCEC apoptosis, rather than by significantly reducing tear secretion (4). High serotonin levels in the tear fluid of DED individuals is the critical cause of serious inflammation and apoptosis in corneal epithelial cells (4, 22). Serotonin receptors are abundant in corneal epithelial cells, and 5-HT acts through serotonin receptors to activate NF-κB signaling (35). Activated NF-κB regulates the transcription of downstream genes and induces a rapid inflammatory response, cell apoptosis, and innate and adaptive immune functions (36). The results systematically confirmed that the presence of serotonin in HCECs and the activation of the NF-κB pathway were important etiological factors for DED. The present findings also suggested that treatment with WAY-100635 was associated with fewer inflammatory cytokines and chemokines.

Collectively, these results suggested that the 5-HT_1A_ receptor-mediated activation of ROS signaling pathways lead to impaired autophagy. The identification of the molecular mechanism may help clarify the pathogenesis and treatment of DED.

## Data Availability Statement

The datasets presented in this study can be found in online repositories. The names of the repository/repositories and accession number(s) can be found in the article/Supplementary Material.

## Ethics Statement

The animal study was reviewed and approved by all experimental protocols complied with the Association for Research in Vision and Ophthalmology Staement for the use of animals and were approved by the Fudan University Ethics Committee (ethical code: EENTIRB-2018-03-01).

## Author Contributions

XZ performed the *in vivo* experiments. YD and ZZ analyzed the data. XZ and JH designed and supervised the project and wrote the manuscript. All authors contributed to the article and approved the submitted version.

## Funding

This work was financially supported by the National Natural Science Foundation of China (81970766 and 82171102), the Program for Professor of Special Appointment (Eastern Scholar) at Shanghai Institutions of Higher Learning, the Shanghai Innovation Development Program (2020-RGZN-02033), the Shanghai Key Clinical Research Program (SHDC2020CR3052B), and the Natural Science Foundation of Shanghai (19ZR1408400).

## Conflict of Interest

The authors declare that the research was conducted in the absence of any commercial or financial relationships that could be construed as a potential conflict of interest.

## Publisher's Note

All claims expressed in this article are solely those of the authors and do not necessarily represent those of their affiliated organizations, or those of the publisher, the editors and the reviewers. Any product that may be evaluated in this article, or claim that may be made by its manufacturer, is not guaranteed or endorsed by the publisher.
