# Supplementary material for: WAY-100635 Alleviates Corneal Lesions Through 5-HT1A Receptor-ROS-Autophagy Axis in Dry Eye
Source: Front Med (Lausanne). 2021 Dec 14;8:799949. doi: 10.3389/fmed.2021.799949 (PMC8712493; doi:10.3389/fmed.2021.799949)
Supplement: Supplementary file 1 [file Data_Sheet_1.docx]

Supplementary Material

**WAY-100635 alleviates corneal lesions through 5-HT_1A_ receptor-ROS-autophagy axis in dry eye**

Xujiao Zhou^1^, Yiqin Dai^1^, Zimeng Zhai^1^ and Jiaxu Hong^1,2*^

**Correspondence:* Jiaxu Hong

[jiaxu.hong@fdeent.org](mailto:jiaxu.hong@fdeent.org)

Supplementary Figure


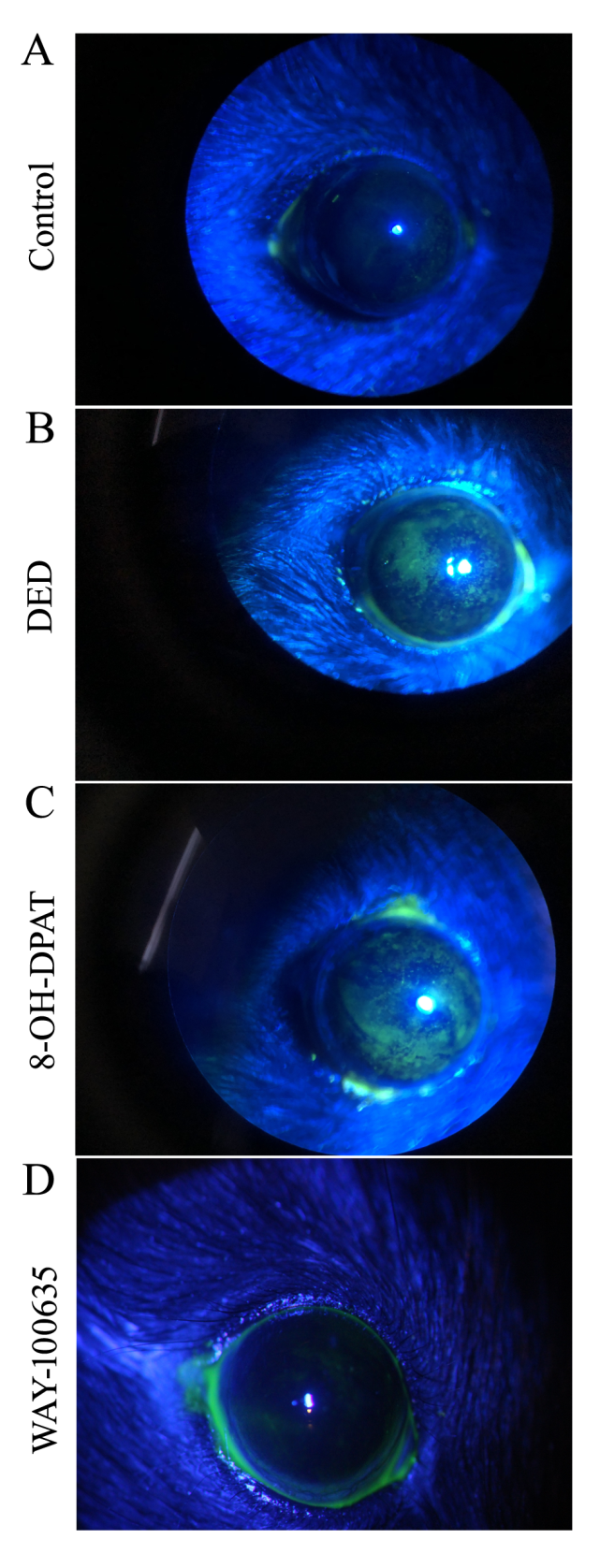


**Supplementary Figure S1.** Original image taken under a Zeiss microscope after staining with sodium fluorescein. **(A)** Control corneal imaging. **(B)** DED corneal imaging. **(C)** Corneal imaging of the 8-OH-DPAT treatment group. **(D)** Corneal imaging of the WAY-100635 treatment group.


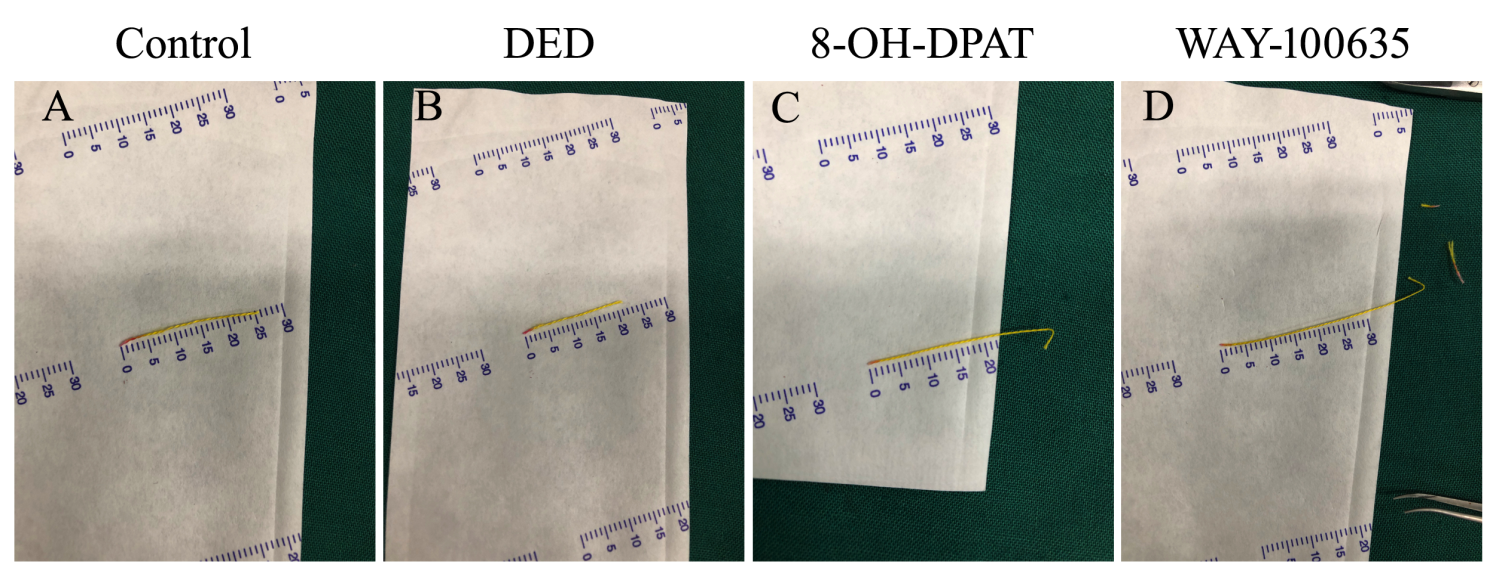


**Supplementary Figure S2.** Original image taken by Phenol-Red thread tear test strip. **(A)** Control imaging. **(B)** DED group imaging. **(C)** Imaging of the 8-OH-DPAT treatment group. **(D)** Imaging of the WAY-100635 treatment group.


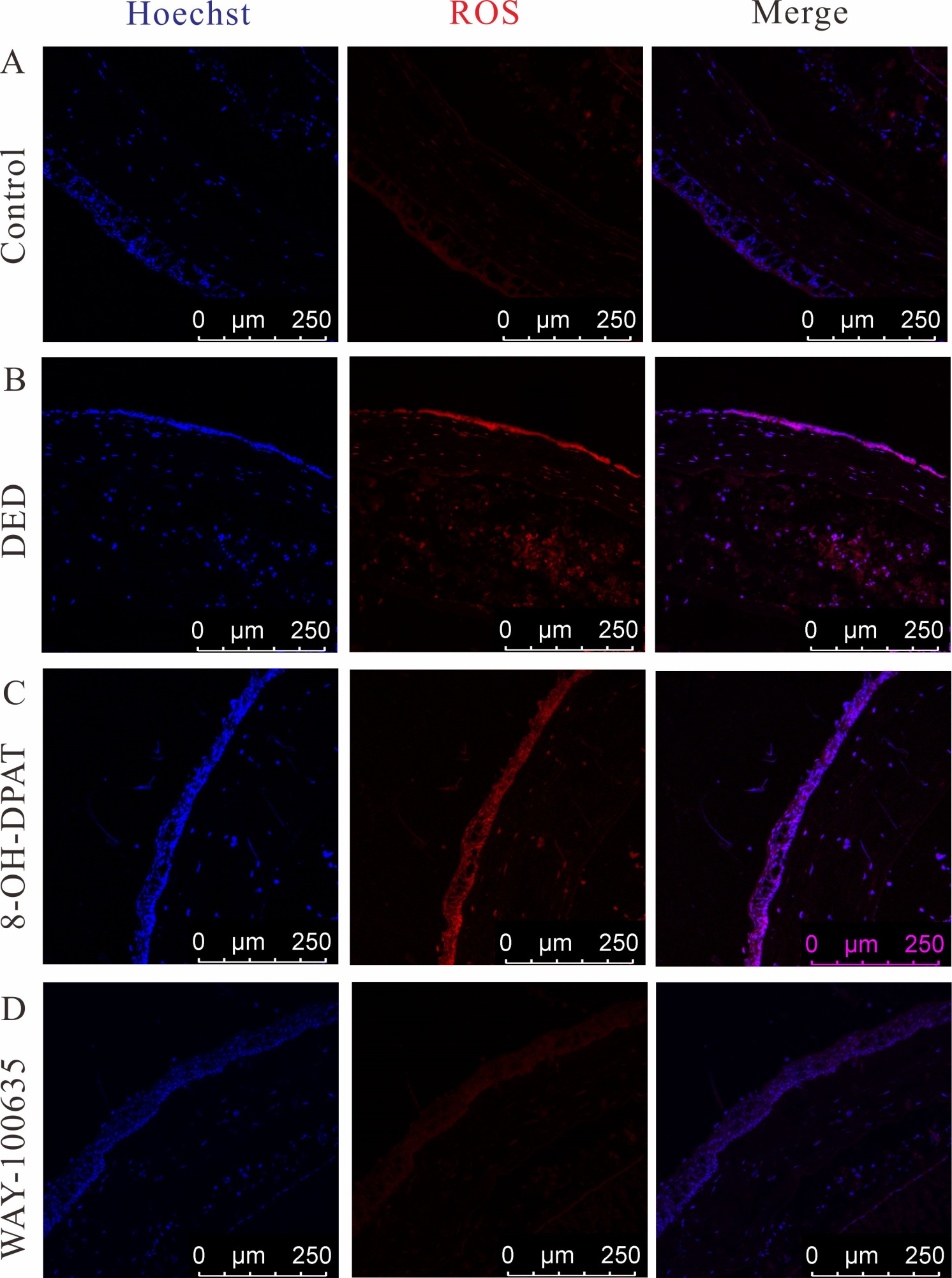


**Supplementary Figure S3.** Original confocal photomicrograph of laser scanning for ROS expression in frozen sections of cornea. **(A)** Control group. **(B)** DED group. **(C)** Corneal confocal imaging of the 8-OH-DPAT treatment group. **(D)** Corneal confocal imaging of the WAY-100635 treatment group. Bar, 250 μm.


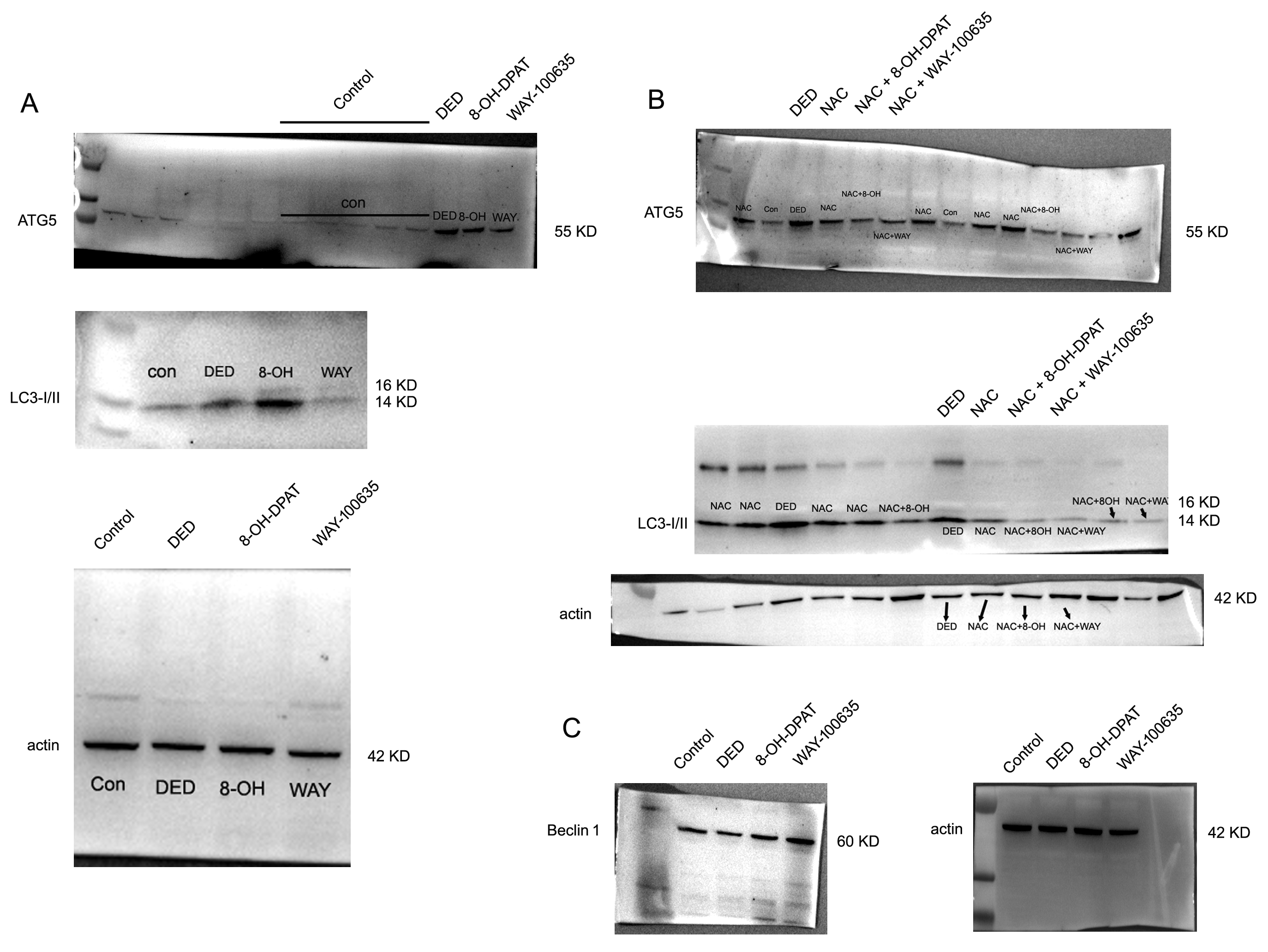


**Supplementary Figure S4.** Full-length blots of Figure 4A and Figure 4D. **(A)** Western blotting analysis of protein expression in control and DED corneas before and after the injection of drugs. Full-length blots of Figure 4A, showing the expression of ATG5 and LC3-I/II. **(B)** Full-length western blot showing the ATG5 and LC3-I/II protein level in DED, NAC, NAC + 8-OH-DPAT and NAC + WAY-100635 groups. **(C)** Full-length western blot showing the Beclin 1 protein level in control, DED, 8-OH-DPAT and WAY-100635 groups.


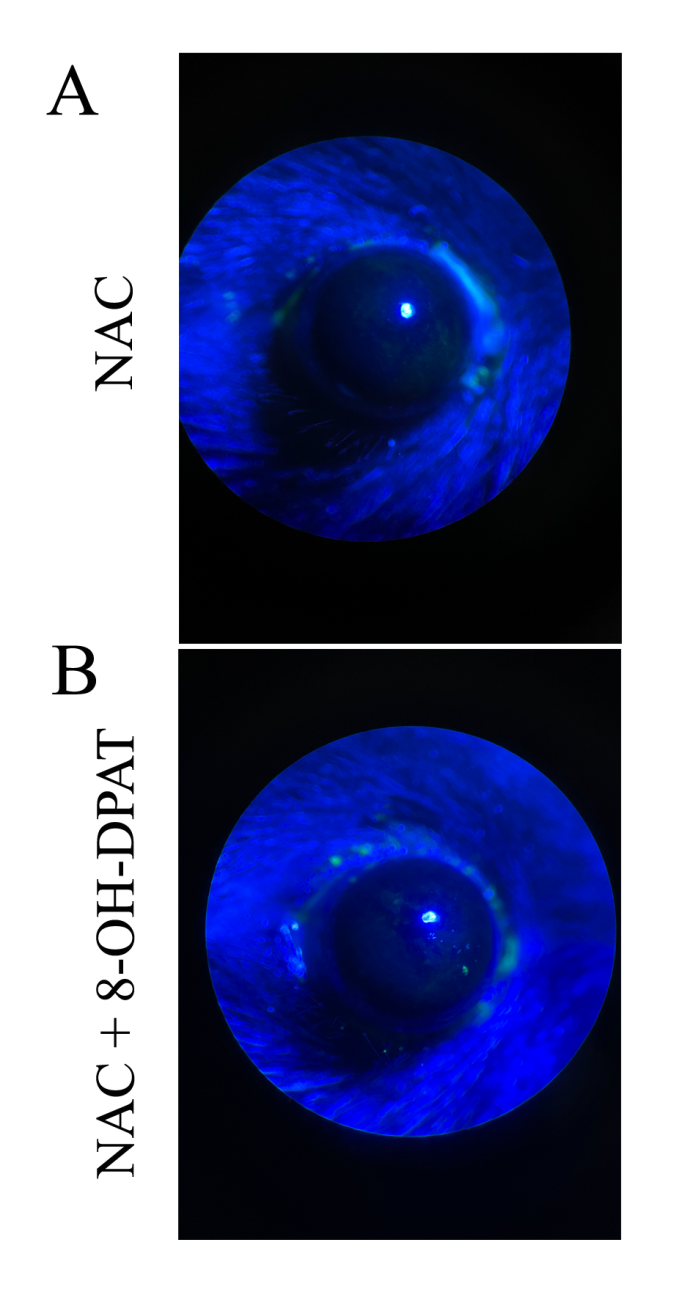


**Supplementary Figure S5.** Original image taken under a Zeiss microscope after staining with sodium fluorescein. **(A)** Corneal imaging of NAC group. **(B)** Corneal imaging of the NAC + 8-OH-DPAT treatment group.
